# Warming sea surface temperatures are linked to lower shorebird migratory fuel loads

**DOI:** 10.1098/rsos.240324

**Published:** 2024-07-17

**Authors:** Benjamin J. Lagassé, Greg A. Breed

**Affiliations:** ^1^ Department of Biology and Wildlife, University of Alaska Fairbanks, Fairbanks, AK, USA; ^2^ Institute of Arctic Biology, University of Alaska Fairbanks, Fairbanks, AK, USA

**Keywords:** climate change, fuel load, intertidal wetland, migratory performance, sea surface temperature, shorebirds

## Abstract

Warming sea surface temperatures (SSTs) are altering the biological structure of intertidal wetlands at a global scale, with potentially serious physiological and demographic consequences for migratory shorebird populations that depend on intertidal sites. The effects of mediating factors, such as age-related foraging skill, in shaping the consequences of warming SSTs on shorebird populations, however, remain largely unknown. Using morphological measurements of Dunlin fuelling for a >3000 km transoceanic migration, we assessed the influence of climatic conditions and age on individuals’ migratory fuel loads and performance. We found that juveniles were often at risk of exhausting their fuel loads en route to primary wintering grounds, especially following high June SSTs in the previous year; the lagged nature of which suggests SSTs acted on juvenile loads by altering the availability of critical prey. Up to 45% fewer juveniles may have reached wintering grounds via a non-stop flight under recent high SSTs compared to the long-term trend. Adults, by contrast, were highly capable of reaching wintering grounds in non-stop flight across years. Our findings suggest that juveniles were disproportionately impacted by apparent SST-related declines in critical prey, and illustrate a general mechanism by which climate change may shape migratory shorebird populations worldwide.

## 1. Introduction

Warming sea surface temperatures (SSTs) and increasingly variable precipitation patterns are altering species interactions at global scales. These new interactions are impacting physiological conditions and processes of individuals that scale up to shift population demographic trends [1,2]. In seasonal environments, for example differences in phenological adjustments among trophic levels [3,4] may result in temporal mismatches between peak consumer demand and peaks in prey resources, asynchronies which may negatively affect consumer body condition and survival [5,6]. Altered seasonal climatic conditions may also alter species’ distributions, levels of inter- and intraspecific competition and mutualistic interactions [2,7].

The magnitude and direction of effects of climate-altered species interactions on populations, however, often depend on mediating biotic and abiotic factors [2]. Differences in foraging experience and skill, for example, may facilitate higher intake rates in older individuals, mitigating negative effects of climate-induced prey shortages on age-specific survival rates [8,9]. Understanding how biotic and abiotic factors interact to shape the consequences of current climatic conditions, and interannual variation, may thus inform predictions of future species interactions and demographic trends under ongoing climate change [10,11].

One key and widespread impact of climate change is the extent to which migratory populations are faced with short- and long-term changes in prey availability across their annual ranges [5,12]. Arctic and subarctic-breeding shorebirds (Scolopacidae and Charadriidae) for example perform some of the longest and most extreme migrations among terrestrial vertebrates. Non-stop migratory flights regularly exceed 3000 km and involve the crossing of barriers such as oceans and deserts with little opportunity of safely stopping *en route* [13]. To conduct these long-distance flights, shorebirds employ a ‘staging’ migration strategy, whereby large proportions of populations congregate annually at specific sites that have uniquely predictable and abundant prey resources, allowing individuals to attain high fuelling rates and sufficiently large fuel loads [14,15].

The dependence of staging shorebird populations on key sites, and the high-quality prey therein, represents a potential bottleneck whereby human- or climate-induced changes in site or prey availability may result in poor body conditions across entire populations, low survival rates and steep population declines [16–19]. Despite the importance of site conditions and juvenile and adult survival rates in driving shorebird population trends [20], the effects of staging-site climatic conditions on specific age classes remain largely unknown [12] (but see [21]). Quantifying the extent to which climatic conditions affect the physiological status of juveniles and adults fuelling for migratory flights could reveal important mechanisms by which climate change could drive shorebird demographic trends [10,11].

The Yukon–Kuskokwim Delta (YKD) in western Alaska represents a potential bottleneck for many Arctic and subarctic shorebird populations in the North Pacific. Intertidal habitats in the YKD are key staging grounds for over a million individuals during southbound migration, including multiple significant staging populations fuelling for non-stop, transoceanic flights to wintering grounds in Australasia and the Pacific coast of North America [22,23]. With ongoing climate change, the Bering Sea region of the YKD is experiencing warming SSTs, longer, more intense marine heatwaves and changes in seasonal sea ice extent and patterns of marine primary production that are likely affecting the high-quality prey upon which staging shorebird populations rely [24–27]. Unlike many of the world’s intertidal sites, the YKD is relatively free of human-induced habitat loss and degradation [28] providing an unique opportunity to identify the extent to which annual climatic conditions may affect the physiological status of juvenile and adult shorebirds preparing for long-distance migratory flights, independent of direct human-induced habitat changes.

Here, using morphological measurements of Dunlin *Calidris alpina* captured in Angyoyaravak Bay, YKD (9 years of captures between 1978 and 2010), we assessed the influence of annual climatic conditions on fuel loads of juveniles and adults in the weeks preceding initiation of southbound migration. We then employed a deterministic flight performance model to assess the relative effects of variation in migratory fuel loads on individuals’ ability to undergo the population’s preferred migratory route—a >3000 km transoceanic migration from the YKD to the northern extent of the species’ primary wintering grounds on the Pacific coast of North America [29–31]. Taken together, we assessed the extent to which annual climatic conditions may affect age-specific migratory performance. Specifically, we ask if climate change is lowering migratory fuel loads such that migrants are less likely to reach primary wintering grounds via a non-stop flight, and whether these impacts are disproportionately being borne by juveniles or adults. Thereby, we assess a general mechanism by which climate change may shape migratory shorebird populations worldwide.

## 2. Methods

### 2.1. Study population

**Figure 1.**
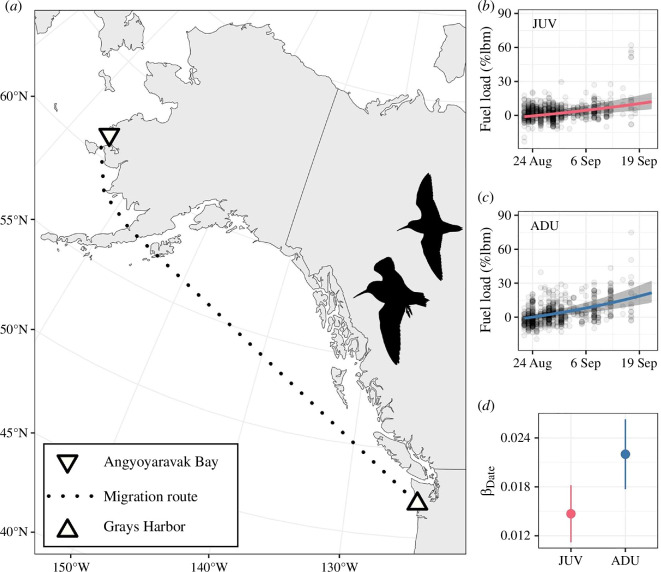
(*a*) Location where Dunlin were captured (Angyoyaravak Bay) and the minimum migratory distance between the YKD, Alaska, and the northern extent of the species’ primary wintering grounds on the Pacific coast of North America (Grays Harbor, Washington). Temporal change in mean fuel loads of (*b*) juvenile (JUV; *n* = 590) and (*c*) adult (ADU; *n* = 590) Dunlin captured during the southbound migratory fuelling period (22 August−22 September 1978−1980, 2008−2010) in Angyoyaravak Bay. Fuel loads (i.e. the amount of residual lean mass and fat individuals were carrying at the time they were captured and weighed, after correcting for differences in individuals’ structural size [% structural lean body mass; %lbm]) were estimated using a linear mixed model (see text). Shaded areas represent 95% confidence intervals (CIs). (*d*) Mean log-fuel accumulation rate (log[%lbm]/day; ±95% CI) by age.

The *pacifica* subspecies of Dunlin is a population of small (50−60 g; [32]) migratory sandpiper endemic to the Pacific Americas Flyway [30,33]. Individuals arrive on YKD breeding grounds in early May, coincident with the availability of snow-free habitat [34], and nest on the ground in low-lying wet graminoid meadows from late May to late June [35]. Eggs typically hatch after 21 days of incubation, and chicks fledge at 21−26 days old [36]. In July, fledged juveniles and post-breeding adults relocate to the YKD coast where they feed extensively on *Limecola balthica* clams available in the region’s vast intertidal mudflats [37], and moult their body feathers (juveniles and adults) and the previous year’s flight feathers (adults only; [38]). By late August, more than 100 000 juvenile and adult Dunlin have relocated to the YKD coast [22], completed or nearly completed their moult [38], and begun fuelling for a >3000 km migration across the Gulf of Alaska to wintering grounds on the Pacific coast of North America (figure 1; [29–31]). During the fuelling period, a portion of the *arcticola* subspecies of Dunlin that breeds in northern Alaska and winters in East Asia also stages in the YKD [30,39]. Juveniles and adults stage into late September and early October and depart synchronously [22,31], with heavier individuals tending to depart 2.5 days earlier per additional gram of body mass at time of capture [31].

Juvenile and adult Dunlin were captured between 1 August and 4 October 1978−1980, 2004−2006 and 2008−2010 in Angyoyaravak Bay, YKD (figure 1) using propelled nets (rocket or spring-loaded), mist nets and walk-in traps placed at or near high-tide roosts along the immediate coast [40]. Upon capture, each bird received an US Geological Survey metal band, had its morphological measurements taken (body mass to the nearest 0.5 g [1978−1980, 2004] or 0.1 g [2005−2010], exposed culmen to the nearest 0.1 mm) and was aged by plumage (juvenile or adult; [37]).

### 2.2. Statistical analyses

#### 2.2.1. Pre-migratory body condition

Dunlin body mass varies among and within individuals at different points in the year. Part of the variation in mass is owing to: (i) differences in individuals’ structural size [32], and part to (ii) individuals seasonally gaining and losing fat and fat-free mass (i.e. lean body mass) in response to life history demands (e.g. migration; [37]). To assess annual variation in individuals’ migratory fuel loads—i.e. the amount of residual lean mass and fat individuals were carrying at the time they were captured and weighed—we therefore had to first control for differences in individuals’ structural size, or structural lean body mass—i.e. the mass of individuals’ structural body components (e.g. skeleton and respiratory system)—and control for differences in individuals’ capture dates.

To estimate and control for differences in individuals’ structural lean body masses, we first fitted linear mixed models with mass at capture as the response variable; culmen length, culmen length + age or an interaction between culmen length and age as explanatory variables; and year as a random intercept using the R package lme4 (v. 1.1−34; [41]). We restricted this analysis to captures that occurred during 1−21 August because this period precedes completion of the pre-basic moult and is before fuelling for southbound migration begins, thereby allowing us to assume that captured individuals had minimal amounts of residual lean mass and fat [38,42]. We chose to regress mass by culmen length because other morphological measurements were not always collected, and because previous studies show that culmen length alone is highly correlated with structural size across Dunlin subspecies and sexes [42,43]. We included age as an explanatory variable to account for potential differences between recently born juveniles and adults in culmen length–structural lean body mass relationship. Finally, we restricted this analysis and all other analyses to captures of individuals with a culmen length >31.5 mm, thus removing males of the *arcticola* subspecies (<2% of captures) that have a structural lean body mass–culmen length relationship different than that of individuals of the *pacifica* subspecies [32,33]. Individuals of the *arcticola* subspecies with a culmen length >31.5 mm could not be separated from *pacifica* individuals and were, therefore, included in our analyses. Prior analyses indicated that there was not a difference in fuel loads of individuals of unknown subspecies (i.e. individuals with a culmen length >31.5 mm and <40.9 mm) and those of female *pacifica* with a culmen length >40.8 mm, indicating that our results are not biased by our inability to remove certain individuals of the *arcticola* subspecies (electronic supplementary material, figure S1).

Using Akaike information criterion (AIC), we found that the linear model containing an interaction between culmen length and age was the most parsimonious (electronic supplementary material, table S1, figure S2). We then used the parameter estimates from this linear model to estimate structural lean body masses and, therefrom, fuel loads of individuals captured during the migratory fuelling period (22 August−22 September). Specifically, we estimated an individual’s migratory fuel load by subtracting their structural lean body mass from their body mass upon being captured. This approach assumed that the culmen length–structural lean body mass relationship identified in pre-fuelling individuals (captured 1−21 August; electronic supplementary material, figure S2) was identical to that of fuelling individuals (captured 22 August−22 September). We standardized estimated fuel loads by dividing an individual’s fuel load by their structural lean body mass. Fuel load estimates, therefore, represent an approximation of the relative fuel load individuals were carrying at the time they were captured and weighed (as a % of their structural lean body mass [%lbm]).

To control for differences in individuals’ capture dates, we next calculated mean daily fuel loads during the migratory fuelling period by fitting gamma-distributed generalized linear mixed models [41] with a log link for juvenile and adult Dunlin using migratory fuel load at capture as the response variable, date as an explanatory variable and year as a random intercept. To ensure juveniles and adults had equal representation across years and sampling periods in these baseline models, we modified the datasets used to fit our models as follows: (i) we divided the migratory fuelling period into four 8 day sampling periods; (ii) we identified the minimum number of age-specific captures per sampling period–year combination; and (iii) we subsampled (without replacement) the age class with the most captures the number of times equal to the sample size of the age class with the least captures. We only included years with >25 captures per age class in our final dataset (*n* = 6 years; 1978−1980 and 2008−2010; electronic supplementary material, table S2). We then estimated individuals’ pre-migratory body conditions—i.e. the extent to which individuals’ fuel loads were above or below mean fuel loads, given individuals’ capture dates—by subtracting the mean fuel load on an individual’s capture date (given our baseline models) from their estimated fuel load. Positive values indicated an above-average fuel load after accounting for an individual’s age, structural size and capture date, and negative values indicated a below-average fuel load [44].

#### 2.2.2. Climatic effects

To explore climatic effects on annual variation in individuals’ migratory fuel loads, we conducted sliding window analyses using the R package climwin (v. 1.2.3; [45,46]). In short, a sliding window analysis systematically explores the influence of a weather variable on a biological response across a range of defined time periods. We used a linear mixed model with pre-migratory body condition as the response variable and year as a random intercept as our baseline model. We then identified the time period candidate weather variables (average daily air temperature [°C; avg air temp], average daily SST [°C; avg SST], avg SST in the previous year [year_*i*-1_], and average daily precipitation [mm; avg precip]) explained the most variation in Dunlin pre-migratory body condition by iteratively adding to the baseline model the mean of a given weather variable within a given candidate climate window, and ranking models using AIC corrected (AICc) for small sample sizes. Among our candidate weather variables, we assessed SSTs from the year before birds were captured, in addition to SSTs in the year of capture, to assess the hypothesis that SSTs in the previous year could affect the availability of critical prey (i.e. 1 year old *L. balthica* clams) and, thereby, affect Dunlin pre-migratory body conditions. This hypothesis is based on the following observations from the YKD and the Wadden Sea region of the North Sea: (i) in Angyoyaravak Bay, YKD, Dunlin fuelling for southbound migration feed extensively on *L. balthica* clams (e.g. 97% of prey items in 38 stomachs examined; [37]); (ii) annual recruitment of 0 year old *L. balthica* clams varies widely between years and is associated with temperature-driven variation in predator abundances and timing of phytoplankton blooms [47,48]; (iii) recruitment of 0 year old *L. balthica* clams drives the subsequent availability of 1 year old clams [49,50]; and (iv) small (1 year old) *L. balthica* clams have greater energetic value and are preferentially eaten at higher rates by a closely related shorebird species than larger (older) *L. balthica* clams [51,52]. We allowed the start and end dates of candidate climate windows to vary (or ‘slide’) at weekly intervals across pre-breeding, breeding and molting periods (1 May−21 August; candidate window range: 1−17 weeks in duration), thereby capturing the environmental and biological processes that likely affect Dunlin fuel loads, while also assessing identical climatic conditions across our sample of individuals and reducing the effects of anomalous weather events and spurious correlations that may occur over short time periods [46,53]. We fitted candidate models with linear and quadratic response functions, and considered models with ΔAICc < 2 to have equal support.

Comparing a large set of candidate models may lead to spurious results and uncertainty in selecting a best model because many models may have similar levels of support [45,46]. To assess the likelihood that top models represented spurious false-positive results, we used the climwin randomization function to compare observed levels of model support with levels of model support from 10 randomized versions of our dataset. We considered models with a *P*_C_ value <0.10 to represent a real climate signal [46]. We then built a global model, and all possible subset models (i.e. ‘all subsets’ models), with pre-migratory body condition as the response variable, influential weather variables and second-order interactions as explanatory variables, and year as a random intercept. We assessed collinearity between influential weather variables using Pearson’s product–moment correlations. We excluded subset models with correlated linear terms (*r* > 0.7) but retained models with correlated interaction terms. Ultimately, our ‘all subsets’ approach identified a single model, with just one influential weather variable (SST year_*i*-1_), that performed substantially better than all other models (i.e. ΔAICc > 2). We then returned to the ‘sliding window’ model set (see above) for the weather variable in the top all subsets model and averaged candidate models that had an Akaike model weight > 0.05 (i.e. the top all subsets model and corresponding sliding window models with an Akaike model weight > 0.05). Daily air and SSTs and precipitation values for the central YKD (60.5°−62° N, 164.75°−166.75° W; hourly data on single levels; 0.25° × 0.25° gridded spatial resolution) were acquired from The European Center for Medium-Range Weather Forecasts ERA5 reanalysis data set [54]. We restricted analyses of climatic effects to juveniles because adult sample sizes were not adequate for conducting sliding window analyses (i.e. there were <9 years with >25 captures per year; electronic supplementary material, table S3; [55]).

#### 2.2.3. Flight range predictions

To understand how annual variation in individuals’ migratory fuel loads might affect individuals’ migratory performance, we conducted flight range simulations using program Flight [56,57] implemented in the R package FlyingR (v 0.2.2; [58]). In short, program Flight combines a biomechanical flight model with a bird’s physical and physiological characteristics to predict its maximum flight range (electronic supplementary material, table S4). One key variable in flight range prediction is departure fuel load—i.e. residual lean mass and fat upon departure. We predicted juvenile and adult departure fuel loads using capture records from early October; the period of the year when individuals are at their heaviest and most likely to depart on southbound migration [22,37]. Specifically, we drew 100 departure fuel loads for juveniles and adults from normal distributions with means and standard deviations informed by October capture records (*n* = 5 and 5, respectively; electronic supplementary material, table S3). Although limited, the number of October capture records we used represent, to the best of our knowledge, the only available information on near-departure body masses of Dunlin from the YKD. Because all October captures occurred in the same year (1980), we then adjusted departure fuel loads according to variation in mean annual pre-migratory body conditions and mean climatic effects identified in sliding window analyses (electronic supplementary material, table S5). This approach assumed: (i) that body conditions during the fuelling period reflected body conditions at departure, and (ii) departure timing was fixed (independent of body condition), both of which are simplifications of complex processes. These simplifications were necessary for assessing the influence of climatic conditions and age on individuals’ ability to undergo the population’s preferred migratory route. The potential impact of these assumptions on interpretation is addressed in §4.

Another key factor in flight range prediction is wind assistance, as Dunlin likely time their southbound migration to coincide with supportive wind conditions [37]. We accounted for wind assistance in our flight range predictions following a 3-step process. First, we calculated the average bearing of the shortest route to the population’s primary wintering grounds (i.e. 112.5°; electronic supplementary material, figure S3). Second, we calculated average daily wind assistance values (given a bearing of 112.5°) for the entire migratory corridor (spanning ~250 km on either side of the shortest migratory route; electronic supplementary material, figure S3) from 29 September to 11 October 1978−1980, 2004−2006 and 2008−2010 [37]. Average daily wind assistance values were estimated at an altitude of ~750 m.a.s.l. (i.e. at the 925 hPa atmospheric pressure level; [59,60]) using the NCEP.Airspeed function in the R package RNCEP (v. 1.0.10; [61]). The equation we used assumed that individuals maintained a constant airspeed (16.1 ms^−1^; [62]) and completely compensated for lateral drift [61,63]. Daily wind values (hourly data at the 925 hPa pressure level; 0.25° × 0.25° gridded spatial resolution) were acquired from The European Center for Medium-Range Weather Forecasts ERA5 reanalysis dataset [54]. Finally, we accounted for wind assistance by setting the average airspeed of our flight range predictions equal to Dunlins’ average speed under still air conditions (16.1 ms^−1^; [62]) plus the 80th percentile of wind assistance values (5.4 ms^−1^; electronic supplementary material, figure S3; i.e. 21.5 ms^−1^). Accounting for wind assistance in this way required that, in program Flight, we set the ‘airspeed–minimum power speed at start’ ratio to 1.98 (electronic supplementary material, table S4; [62]). All analyses were conducted using program R (v. 4.3.1; [64]).

## 3. Results

A total of 2352 juvenile and 1828 adult Dunlin were captured in Angyoyaravak Bay between 1 August and 4 October 1978−1980, 2004−2006 and 2008−2010. Captures ranged from 71 to 467 juveniles and 1−858 adults per year, with 3−100% of captures occurring during migratory fuelling periods (22 August−22 September; electronic supplementary material, table S3). Pre-migratory body conditions did not differ by capture method (electronic supplementary material, figure S4).

### 3.1. Pre-migratory body condition

**Figure 2.**
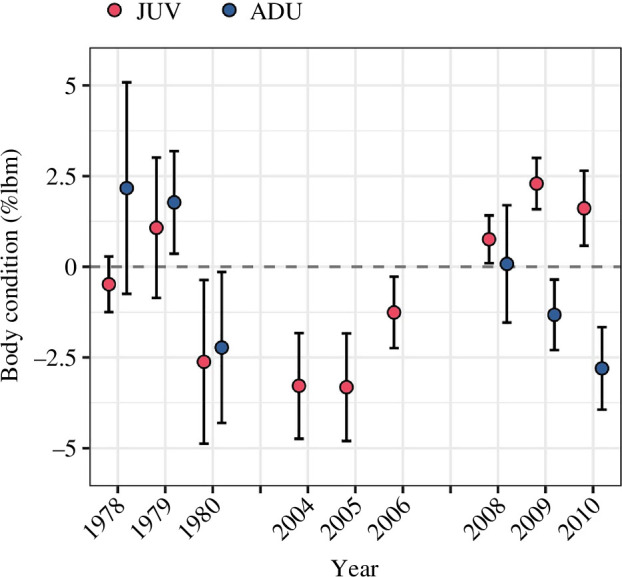
Mean annual pre-migratory body condition of juvenile (JUV; *n* = 1459) and adult (ADU; *n* = 1133) Dunlin (±95% CI) captured during the southbound migratory fuelling period (22 August−22 September) in Angyoyaravak Bay, YKD, Alaska. Pre-migratory body conditions (i.e. the extent to which fuel loads were above or below average after correcting for individuals’ age, structural size and capture date [% structural lean body mass; %lbm]) were estimated using age-specific linear and generalized linear mixed models and, therefore, are not equivalent between juveniles and adults (see text and figure 1).

Juvenile and adult Dunlin had similar fuel loads when they began fuelling for southbound migration (22 August; mean: −1.18% of structural lean body mass [%lbm; 95% CI: −3.19, 1.03] for juveniles and −1.00 %lbm [−2.74, 0.89] for adults). Adults, however, tended to have a higher accumulation rate (β_Date_; 0.0147 log[%lbm]/day [0.0112, 0.0182] for juveniles and 0.0220 log[%lbm]/day [0.0177, 0.0263] for adults; figure 1). Mean annual pre-migratory body conditions ranged from −3.32 to 2.29 %lbm and −2.80 to 2.17 %lbm for juveniles (*n* = 9 years) and adults (*n* = 6 years), respectively (figure 2).

### 3.2. Climatic effects

**Figure 3.**
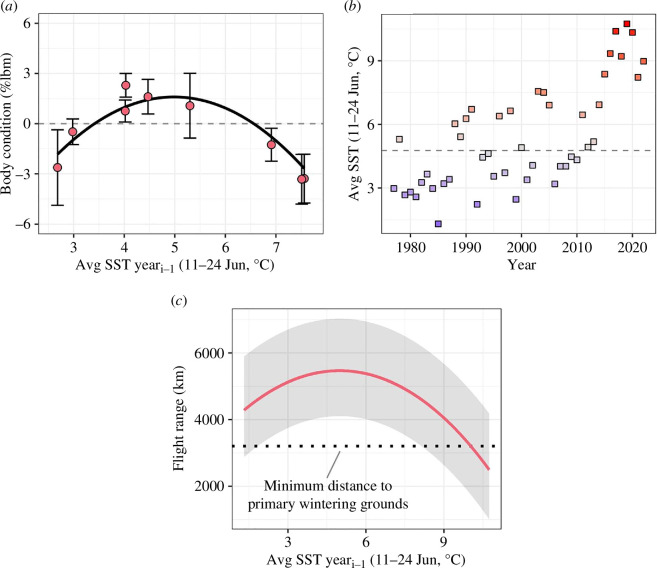
(*a*) Effect of average SST (11−24 June) in the previous year (year_*i*-1_) on juvenile Dunlin (*n* = 1459) pre-migratory body condition (mean ± 95% CI) during the southbound migratory fuelling period (22 August−22 September 1978−1980, 2004−2006, 2008−2010) in Angyoyaravak Bay, YKD, Alaska. Pre-migratory body conditions (i.e. the extent to which fuel loads were above or below average after correcting for individuals’ age, structural size and capture date [% structural lean body mass; %lbm]) were estimated using linear and generalized linear mixed models (see text). (*b*) Average SST in the central YKD by year. The horizontal line represents the median annual SST value from 1977 to 2022. (*c*) Mean effect of average SST year_*i*-1_ on median predicted flight ranges of juvenile Dunlin departing Angyoyaravak Bay on southbound migration (1978−2022). The shaded area represents the interquartile range of predicted flight ranges. Flight range predictions were generated using program Flight and account for wind assistance (see text).

Average SST in the previous year (SST year_*i*-1_ 11–24 June) and average precipitation in the year of capture (4 June−29 July) were influential predictors of juvenile pre-migratory body condition and were negatively correlated with each other during our study (*r* = −0.87). For both weather variables, quadratic response functions fit better than linear responses (electronic supplementary material, table S6). Comparison of the top model for each weather variable and a model including their interaction suggested SST in the previous year (SST year_*i*-1_ 11–24 June) alone was the best predictor of juvenile pre-migratory body condition (electronic supplementary material, table S7). Four SST year_*i*-1_ sliding window models had Akaike model weights >0.05 and were averaged (electronic supplementary material, table S8). Intermediate SST year_*i*-1_ values were associated with above-average pre-migratory body conditions, and high SST year_*i*-1_ values were associated with the lowest average body conditions (figure 3).

### 3.3. Flight range predictions

**Figure 4.**
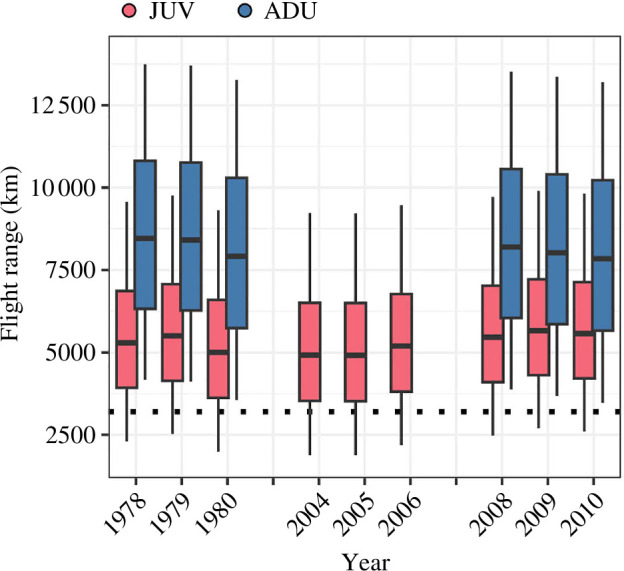
Median predicted flight ranges of juvenile (JUV) and adult (ADU) Dunlin departing Angyoyaravak Bay, YKD, Alaska, on southbound migration. Boxes and whiskers represent the inner 50% and 90% of predicted flight ranges. Flight range predictions were generated using program Flight and account for wind assistance (see text). The horizontal dotted line represents the minimum distance between Angyoyaravak Bay and the northern extent of the species’ primary wintering grounds on the Pacific coast of North America (see figure 1).

Predicted fuel loads for Dunlin departing Angyoyaravak Bay were smaller for juveniles (median range across years: 34−39 %lbm [interquartile range across years; IQR: 24−51]; *n* = 9 years) than for adults (56−61 %lbm [IQR: 39−81]; *n* = 6 years), and resulted in flight range predictions for adults being 2100−3600 km greater (7842−8460 km [IQR: 5664−10 812]) than juveniles’ (4912−5664 km [IQR: 3522−7221]). Juvenile and adult flight range predictions were generally adequate for reaching primary wintering grounds on the Pacific coast of North America, but adults had greater surplus fuel loads (difference between median flight ranges and minimum migration distance [shortest migration route = 3205 km]; juveniles: 1707−2459 km, adults: 4637−5255 km; figure 4). Juvenile median flight range predictions under long-term (1978−2022) SST year_*i*-1_ conditions ranged from 2493 to 5470 km and were adequate for reaching primary wintering grounds on the Pacific coast of North America under typical long-term climatic conditions. Median flight range predictions fell below the minimum migration distance, however, when SSTs were much warmer (>10.0°C) than climatological means. Up to 45% fewer juveniles may have reached primary wintering grounds via a non-stop flight under recent high SSTs compared to the long-term trend (i.e. 10.3°C versus 4.8°C; figure 3).

## 4. Discussion

Warming SSTs are altering the biological structure of intertidal wetlands [65] with potentially serious physiological and demographic consequences for migratory shorebird populations that depend on intertidal sites [16,18]. Using body mass measurements of Dunlin fuelling for a >3000 km transoceanic migration from the YKD, Alaska, to wintering grounds on the Pacific coast of North America, we show that juvenile pre-migratory body conditions were correlated with June SSTs in the previous year, with high June SSTs driving low migratory fuel loads the following year. The clear lagged nature of the observed effect suggests SSTs likely acted on fuel loads by altering the availability of critical prey. We propose that the lagged effect reflects a link between SSTs and the recruitment of *L. balthica* clams.

*Limecola balthica* clams are eaten extensively by Dunlin fuelling in the YKD (e.g. 97% of prey items in 38 stomachs examined; [37]). 1 year old *L. balthica* clams have high energetic value and are likely selected by Dunlin at higher rates than larger (older) *L. balthica* clams [51,52]. Studies suggest the availability of 1 year old *L. balthica* clams is driven by recruitment of 0 year old clams in the previous year [49,50], which varies widely according to temperature-driven variation in predator abundances [47] and timing of *L. balthica* spawning activity in relation to spring phytoplankton blooms [48]. In the western Wadden Sea region of the North Sea, for example high SSTs are associated with earlier spawning activity of *L. balthica* clams, creating a mismatch between spawning and spring phytoplankton blooms. Reduction in phytoplankton available to clam larvae caused by this phenological mismatch is believed to limit food and delay the age and decrease the size at which larvae metamorphose into bottom-dwelling recruits, increasing the overall mortality of 0 year old clams [48, 66].

In the Bering Sea region of the YKD, June SST is correlated with the timing of spring sea ice retreat and phytoplankton blooms [25,67]. In cold years, late ice retreat leads to early ice-associated blooms in cold water (i.e. May), whereas warm years lead to early ice retreat and later blooms in warm water (i.e. June [25,68]; see also [69]). High SSTs and late phytoplankton blooms (i.e. June bloom productivity) are likely better aligned with spawning activity of *L. balthica* clams than the low SSTs early blooms (electronic supplementary material, figure S5). Despite the possibility of a better match between spawning activity and phytoplankton blooms under high SSTs, studies suggest less late-season phytoplankton ultimately enters the benthic food web owing to higher rates of grazing by zooplankton compared to years with colder SSTs and earlier blooms [26,68] (see also [70]). High SSTs in the YKD, therefore, may result in less phytoplankton available to developing larvae and 0 year old *L. balthica* clams, decreasing larval recruitment, and subsequently limiting Dunlin fuelling for southbound migration the following year by reducing the availability of 1 year old clams [71]. Particularly cold (i.e. very cold) SSTs could also reduce the availability of 1 year old clams via later ice retreat, poorer blooming conditions and comparably weaker phytoplankton blooms [26], suggesting the observed quadratic effect of intermediate (i.e. cold) SSTs being associated with above-average fuel loads.

The YKD, and the high-quality prey therein, represents a potential bottleneck for many southbound-migrating shorebird populations in the North Pacific [22,72]. Changes in availability of critical prey within the YKD, such as 1 year old *L. balthica* clams, could alter competitive interactions between shorebird species, sexes and age classes and have far-reaching population-level effects. For example, when critical prey resources initially become scarce, better competitors (i.e. adults with better foraging skills) are usually able to acquire adequate resources, whereas poorer competitors (i.e. juveniles) are often disproportionately impacted [8,9]. Through an increase in juveniles failing to recruit to the breeding population, changes in prey availability can have a significant effect on population structure and size [20,73]. Among Dunlin in the YKD, we found that juveniles and adults had similar fuel loads at the onset of the migratory fuelling period, but adults attained higher fuelling rates and larger migratory fuel loads. Interannual differences in juvenile fuel loads were in turn explained by June SSTs in the previous year. Taken together, these findings suggest that juveniles were outperformed and/or excluded from preferred feeding areas by adults, and were more likely to suffer negative effects from apparent SST-related declines in critical prey [73].

Consequences of prey limitation can be direct, such as immediate starvation, or indirect such as individuals lacking the fuel loads required to migrate in an optimal way. We generated maximum flight range predictions using body mass measurements that accounted for annual variation in migratory fuel loads and found that adults were highly capable of departing the YKD, crossing the Gulf of Alaska, and reaching primary wintering grounds in non-stop flight, whereas juveniles were often at risk of exhausting their fuel loads en route, especially following high June SSTs in the previous year. Poorer foraging skills and/or exclusion from preferred feeding areas, therefore, may have caused a portion of southbound-migrating juveniles to migrate at the edge of their physical ability. Moreover, relatively small changes in prey availability in the YKD, such as possible reductions in *L. balthica* clams with ongoing climate change [74,75], may push juveniles to migrate at the edge of their ability at higher rates (e.g. [76]).

The warmest years of our study (2003−2005) coincided with record late sea ice formation and early spring sea ice retreat in the Bering Sea region of the YKD [67]. Recent significant marine heat waves in the region (2017−2020; [24]) have since resulted in record low sea ice extents, likely reductions in ice-associated phytoplankton blooms, northward shifts in the distribution of crab and fish populations, unprecedented seabird die-offs and widespread reproductive failures and marine mammal mortality events [77]; all indications that ongoing climate change in the eastern Bering Sea could drive long-term changes in energy flow and ecosystem structure [26,77,78].

We found that recent high SSTs in the Bering Sea region of the YKD (2017−2020) may have resulted in up to 45% fewer juveniles reaching primary wintering grounds via a non-stop flight. Given that Dunlin show an endogenous drive to take a direct migratory route over the Gulf of Alaska [29], and that there is no evidence of similarly favourable alternative routes [37], this finding suggests that warming SSTs in the YKD with ongoing climate change [27] could trigger higher juvenile mortality during southbound migration and a lower stable Dunlin population size [20,79] (but see [10,80]). There is ample evidence, however, that migratory behaviours are flexible [81] and shorebirds may compensate behaviourally for smaller fuel loads. Later arrival of harsh winter conditions in the YKD with ongoing climate change [24], for example, could allow populations to stage later and compensate for lower fuelling rates and early October fuels loads [82]. Populations may also stop opportunistically at ancillary stopover sites along the Alaska Peninsula and acquire additional fuel [31,83].

The extent to which *juvenile* shorebirds may flexibly adjust their endogenous migration programs, however, is largely unknown [84]. Studies suggest that juvenile birds, in general, modify their endogenous migration programs as they gain experience [84,85], and have especially high levels of mortality during their first migratory flights [85,86]; levels of mortality that may be moderated by annual variation in climatic conditions and fuel loads [76,87] (see also [13]). At a minimum, smaller fuel loads with warming SSTs may push juvenile Dunlin to take a longer, less efficient and possibly more dangerous coastal migration route rather than a direct over-ocean migratory route [21,88,89], which would represent a significant, climate-induced change in their migratory behaviour.

The extent to which staging shorebird populations are negatively impacted by ongoing climate change may largely depend on the ability of juveniles to flexibly adjust their endogenous migration programs to conditions encountered *en route* [81,85]. Given potentially significant consequences of warming SSTs on staging shorebird populations, additional studies are needed to develop a strong empirical understanding of the links between SSTs, intertidal invertebrate population dynamics and shorebird fuel loads and demographic trends.

## Data Availability

The morphological measurements presented in this study are available at the U.S. Geological Survey Alaska Science Center’s Data Repository [90]. Supplementary material, data and code supporting the results of this study are available at Figshare [91].

## References

[1] Ockendon N *et al*. 2014 Mechanisms underpinning climatic impacts on natural populations: altered species interactions are more important than direct effects. Glob. Chang. Biol. **20**, 2221–2229. (10.1111/gcb.12559) 24677405

[2] Tylianakis JM, Didham RK, Bascompte J, Wardle DA. 2008 Global change and species interactions in terrestrial ecosystems. Ecol. Lett. **11**, 1351–1363. (10.1111/j.1461-0248.2008.01250.x) 19062363

[3] Both C, van Asch M, Bijlsma RG, van den Burg AB, Visser ME. 2009 Climate change and unequal phenological changes across four trophic levels: constraints or adaptations? J. Anim. Ecol. **78**, 73–83. (10.1111/j.1365-2656.2008.01458.x) 18771506

[4] Thackeray SJ *et al*. 2016 Phenological sensitivity to climate across taxa and trophic levels. Nature **535**, 241–245. (10.1038/nature18608) 27362222

[5] Lameris TK, van der Jeugd HP, Eichhorn G, Dokter AM, Bouten W, Boom MP, Litvin KE, Ens BJ, Nolet BA. 2018 Arctic geese tune migration to a warming climate but still suffer from a phenological mismatch. Curr. Biol. **28**, 2467–2473.(10.1016/j.cub.2018.05.077) 30033332

[6] Lameris TK *et al*. 2022 Mismatch‐induced growth reductions in a clade of Arctic‐breeding shorebirds are rarely mitigated by increasing temperatures. Glob. Chang. Biol. **28**, 829–847. (10.1111/gcb.16025) 34862835

[7] Whalen MA, Starko S, Lindstrom SC, Martone PT. 2023 Heatwave restructures marine Intertidal communities across a stress gradient. Ecology **104**, e4027. (10.1002/ecy.4027) 36897574

[8] Nevoux M, Weimerskirch H, Barbraud C. 2007 Environmental variation and experience-related differences in the demography of the long-lived black-browed albatross. J. Anim. Ecol. **76**, 159–167. (10.1111/j.1365-2656.2006.01191.x) 17184364

[9] Oro D, Torres R, Rodríguez C, Drummond H. 2010 Climatic influence on demographic parameters of a tropical seabird varies with age and sex. Ecology **91**, 1205–1214. (10.1890/09-0939.1) 20462134

[10] Dybala KE, Eadie JM, Gardali T, Seavy NE, Herzog MP. 2013 Projecting demographic responses to climate change: adult and juvenile survival respond differently to direct and indirect effects of weather in a passerine population. Glob. Chang. Biol. **19**, 2688–2697. (10.1111/gcb.12228) 23606580

[11] Jenouvrier S. 2013 Impacts of climate change on avian populations. Glob. Chang. Biol. **19**, 2036–2057. (10.1111/gcb.12195) 23505016

[12] Lindström Å, Agrell J. 1999 Global change and possible effects on the migration and reproduction of Arctic-breeding Waders. Ecol. Bull. **47**, 145–159.

[13] Conklin JR, Senner NR, Battley PF, Piersma T. 2017 Extreme migration and the individual quality spectrum. J. Avian Biol. **48**, 19–36. (10.1111/jav.01316)

[14] Atkinson PW *et al*. 2007 Rates of mass gain and energy deposition in red knot on their final spring staging site is both time- and condition-dependent. J. Appl. Ecol. **44**, 885–895. (10.1111/j.1365-2664.2007.01308.x)

[15] Warnock N. 2010 Stopping vs. staging: the difference between a hop and a jump. J. Avian Biol. **41**, 621–626. (10.1111/j.1600-048X.2010.05155.x)

[16] Baker AJ *et al*. 2004 Rapid population decline in red knots: fitness consequences of decreased refuelling rates and late arrival in Delaware Bay. Proc. R. Soc. Lond. B **271**, 875–882. (10.1098/rspb.2003.2663) PMC1691665 15255108

[17] Brlík V *et al*. 2022 Survival fluctuation is linked to precipitation variation during staging in a migratory shorebird. Sci. Rep. **12**, 19830. (10.1038/s41598-022-24141-5) 36400908 PMC9674593

[18] Rakhimberdiev E *et al*. 2018 Fuelling conditions at staging sites can mitigate Arctic warming effects in a migratory bird. Nat. Commun. **9**, 4263. (10.1038/s41467-018-06673-5) 30323300 PMC6189115

[19] Studds CE *et al*. 2017 Rapid population decline in migratory shorebirds relying on yellow sea tidal mudflats as stopover sites. Nat. Commun. **8**, 14895. (10.1038/ncomms14895) 28406155 PMC5399291

[20] Weiser EL *et al*. 2020 Annual adult survival drives trends in Arctic-breeding shorebirds but knowledge gaps in other vital rates remain. Condor **122**, duaa026. (10.1093/condor/duaa026)

[21] Anderson AM, Friis C, Gratto-Trevor CL, Harris CM, Love OP, Morrison RIG, Prosser SWJ, Nol E, Smith PA. 2021 Drought at a coastal wetland affects refuelling and migration strategies of shorebirds. Oecologia **197**, 661–674. (10.1007/s00442-021-05047-x) 34657196 PMC8585834

[22] Gill RE, Handel CM. 1990 The importance of Subarctic intertidal habitats to shorebirds: a study of the central Yukon–Kuskokwim Delta, Alaska. Condor. **92**, 709. (10.2307/1368690)

[23] Ruthrauff DR, Pohlen ZM, Wilson HM, Johnson JA. 2021 Bar-tailed Godwits Limosa lapponica in Alaska: revisiting population estimates from the staging grounds. W.S. **128**, 255–264. (10.18194/ws.00251)

[24] Carvalho KS, Smith TE, Wang S. 2021 Bering sea marine heatwaves: patterns, trends and connections with the Arctic. J. Hydrol. (Amst) **600**, 126462. (10.1016/j.jhydrol.2021.126462)

[25] Clement Kinney J, Maslowski W, Osinski R, Lee YJ, Goethel C, Frey K, Craig A. 2022 On the variability of the Bering sea cold pool and implications for the biophysical environment. PLoS One **17**, e0266180. (10.1371/journal.pone.0266180) 35377921 PMC8979450

[26] Grebmeier JM. 2012 Shifting patterns of life in the Pacific Arctic and sub-Arctic seas. Annu. Rev. Mar. Sci. **4**, 63–78. (10.1146/annurev-marine-120710-100926) 22457969

[27] Hermann AJ, Gibson GA, Cheng W, Ortiz I, Aydin K, Wang M, Hollowed AB, Holsman KK. 2019 Projected biophysical conditions of the Bering sea to 2100 under multiple emission scenarios. ICES J. Mar. Sci. **76**, 1280–1304. (10.1093/icesjms/fsz043)

[28] Santos CD, Catry T, Dias MP, Granadeiro JP. 2023 Global changes in coastal wetlands of importance for non-breeding shorebirds. Sci. Total Environ. **858**, 159707. (10.1016/j.scitotenv.2022.159707) 36306834

[29] Åkesson S, Grönroos J, Bianco G. 2021 Autumn migratory orientation and route choice in early and late dunlins Calidris alpina captured at a stopover site in Alaska. Biol. Open. **10**, bio058655. (10.1242/bio.058655) 33913474 PMC8096618

[30] Gill RE, Handel CM, Ruthrauff DR. 2013 Intercontinental migratory connectivity and population structuring of Dunlins from Western Alaska. Condor **115**, 525–534. (10.1525/cond.2013.120127)

[31] Warnock N, Handel CM, Gill, Jr. RE, McCaffery BJ. 2013 Residency times and patterns of movement of postbreeding Dunlin on a Subarctic staging area in Alaska. Arctic **66**, 407–416. (10.14430/arctic4327)

[32] Gates HR, Yezerinac S, Powell AN, Tomkovich PS, Valchuk OP, Lanctot RB. 2013 Differentiation of subspecies and sexes of Beringian Dunlins using morphometric measures. J. Field Ornithol. **84**, 389–402. (10.1111/jofo.12038)

[33] MacLean, SF, Holmes RT. 1971 Bill lengths, wintering areas, and taxonomy of North American Dunlins, Calidris alpina. Auk. **88**, 893–901. (10.2307/4083846)

[34] Ely CR, McCaffery BJ, Gill Jr RE. 2018 Shorebirds adjust spring arrival schedules with variable environmental conditions: four decades of assessment on the Yukon–Kuskokwim Delta, Alaska. In In trends and traditions: avifaunal change in Western North America (eds WD Shuford, RE Gill Jr, CM Handel), pp. 296–311. Camarillo, CA, USA: Western Field Ornithologists. (10.21199/SWB3)

[35] Jamieson SE. 2011 Pacific Dunlin Calidris alpina pacifica show a high propensity for second clutch production. J. Ornithol. **152**, 1013–1021. (10.1007/s10336-011-0691-4)

[36] Holmes RT. 1966 Breeding ecology and annual cycle adaptations of the red-backed sandpiper (Calidris alpina) in Northern Alaska. Condor **68**, 3–46. (10.2307/1365173)

[37] Warnock ND, Gill Jr RE. 2020 Dunlin (Calidris Alpina), version 1.0. In Birds of the world (ed. SM Billerman). Ithaca, NY: Cornell Lab of Ornithology. (10.2173/bow.dunlin.01)

[38] Holmes RT. 1971 Latitudinal differences in the breeding and Molt schedules of Alaskan red-backed sandpipers (Calidris alpina). Condor **73**, 93–99. (10.2307/1366128)

[39] Lagassé BJ *et al*. 2022 Migratory network reveals unique spatial-temporal migration dynamics of Dunlin subspecies along the East Asian-Australasian flyway. PLoS One **17**, e0270957. (10.1371/journal.pone.0270957) 35925977 PMC9352067

[40] Handel CM, Gill Jr RE. 1992 Roosting behavior of premigratory Dunlins (Calidris alpina). Auk **109**, 57–72. (10.2307/4088266)

[41] Bates D, Mächler M, Bolker BM, Walker SC. 2015 Fitting linear mixed-effects models using lme4. J. Stat. Soft. **67**, 1–48. (10.18637/jss.v067.i01)

[42] Goede AA, Nieboer E. 1983 Weight variation of Dunlins Calidris alpina during post-nuptial moult, after application of weight data transformations. Bird. Stud. **30**, 157–163. (10.1080/00063658309476791)

[43] Piersma T, Brederode NE. 1990 The estimation of fat reserves in coastal waders before their departure from Northwest Africa in spring. Ardea **78**, 221–236.

[44] Duijns S, Niles LJ, Dey A, Aubry Y, Friis C, Koch S, Anderson AM, Smith PA. 2017 Body condition explains migratory performance of a long-distance migrant. Proc. Biol. Sci. **284**, 20171374. (10.1098/rspb.2017.1374) 29093218 PMC5698639

[45] Bailey LD, van de Pol M. 2016 Climwin: an R Toolbox for climate window analysis. PLoS One **11**, e0167980. (10.1371/journal.pone.0167980) 27973534 PMC5156382

[46] van de Pol M, Bailey LD, McLean N, Rijsdijk L, Lawson CR, Brouwer L. 2016 Identifying the best climatic predictors in ecology and evolution. Methods Ecol. Evol. **7**, 1246–1257. (10.1111/2041-210X.12590)

[47] Beukema JJ, Honkoop PJC, Dekker R. 1998 Recruitment in *Macoma balthica* after mild and cold winters and its possible control by egg production and shrimp predation. In Recruitment, colonization and physical-chemical forcing in marine biological systems (eds S Baden, L Phil, R Rosenberg, JO Strömberg, I Svane, P Tiselius), pp. 23–34. Dordrecht: Springer Netherlands. (10.1007/978-94-017-2864-5)

[48] Philippart CJM, van Aken HM, Beukema JJ, Bos OG, Cadée GC, Dekker R. 2003 Climate-related changes in recruitment of the bivalve Macoma balthica. Limnol. Oceanogr. **48**, 2171–2185. (10.4319/lo.2003.48.6.2171)

[49] Beukema JJ, Dekker R, Essink K, Michaelis H. 2001 Synchronized reproductive success of the main bivalve species in the Wadden sea: causes and consequences. Mar. Ecol. Prog. Ser. **211**, 143–155. (10.3354/meps211143)

[50] van Der Meer J, Beukema JJ, Dekker R. 2001 Long‐term variability in secondary production of an intertidal bivalve population is primarily a matter of recruitment variability. J. Anim. Ecol. **70**, 159–169. (10.1111/j.1365-2656.2001.00469.x)

[51] Bachelet G. 1986 Recruitment and year-to-year variability in a population of Macoma balthica (L.). Hydrobiologia **142**, 233–248. (10.1007/BF00026762)

[52] Ruthrauff DR, Dekinga A, Gill RE, van Gils JA, Piersma T. 2015 Ways to be different: foraging adaptations that facilitate higher intake rates in a northerly wintering shorebird compared with a low-latitude conspecific. J. Exp. Biol. **218**, 1188–1197. (10.1242/jeb.108894) 25714569

[53] de Zwaan DR, Drake A, Camfield AF, MacDonald EC, Martin K. 2022 The relative influence of cross‐seasonal and local weather effects on the breeding success of a migratory Songbird. J. Anim. Ecol. **91**, 1458–1470. (10.1111/1365-2656.13705) 35426953

[54] Hersbach H *et al*. 2020 The ERA5 global reanalysis. Quart. J. Royal Meteoro. Soc. **146**, 1999–2049. (10.1002/qj.3803)

[55] Bailey LD *et al*. 2022 Bird populations most exposed to climate change are less sensitive to climatic variation. Nat. Commun. **13**, 2112. (10.1038/s41467-022-29635-4) 35440555 PMC9018789

[56] Pennycuick CJ. 2008 Modelling the flying bird. Amsterdam; London: Elsevier.

[57] Pennycuick CJ, Battley PF. 2003 Burning the engine: a time-marching computation of fat and protein consumption in a 5420-km non-stop flight by great knots, Calidris tenuirostris. Oikos **103**, 323–332. (10.1034/j.1600-0706.2003.12124.x)

[58] Masinde B. 2022 FlyingR: simulation of bird flight range. R package version 0.2.2. https://CRAN.R-project.org/package=FlyingR.

[59] Hedenström A, Alerstam T. 1992 Climbing performance of migrating birds as a basis for estimating limits for fuel-carrying capacity and muscle work. J. Exp. Biol. **164**, 19–38. (10.1242/jeb.164.1.19)

[60] Krietsch J, Valcu M, Kempenaers B. 2020 Wind conditions influence breeding season movements in a nomadic polygynous shorebird. Proc. Biol. Sci. **287**, 20192789. (10.1098/rspb.2019.2789) 32075527 PMC7031675

[61] Kemp MU, Shamoun-Baranes J, van Loon EE, McLaren JD, Dokter AM, Bouten W. 2012 Quantifying flow-assistance and implications for movement research. J. Theor. Biol. **308**, 56–67. (10.1016/j.jtbi.2012.05.026) 22683380

[62] Pennycuick CJ, Åkesson S, Hedenström A. 2013 Air speeds of migrating birds observed by ornithodolite and compared with predictions from flight theory. J. R. Soc. Interface. **10**, 20130419. (10.1098/rsif.2013.0419) 23804440 PMC3730693

[63] Linscott JA, Navedo JG, Clements SJ, Loghry JP, Ruiz J, Ballard BM, Weegman MD, Senner NR. 2022 Compensation for wind drift prevails for a shorebird on a long-distance, transoceanic flight. Mov. Ecol. **10**, 11. (10.1186/s40462-022-00310-z) 35255994 PMC8900403

[64] R Core Team. 2023 R: a language and environment for statistical computing. Vienna, Austria: R Foundation for Statistical Computing. See http://www.R-project.org/.

[65] Intergovernmental Panel on Climate Change (IPCC). 2023 Climate change 2022 – impacts, adaptation and vulnerability: working group II contribution to the sixth assessment report of the intergovernmental panel on climate change, 1st edn. Cambridge, UK: Cambridge University Press. (10.1017/9781009325844)

[66] Phillips NE. 2002 Effects of nutrition-mediated larval condition on juvenile performance in a marine mussel. Ecology **83**, 2562–2574. (10.1890/0012-9658(2002)083[2562:EONMLC]2.0.CO;2)

[67] Stabeno PJ, Bell SW, Bond NA, Kimmel DG, Mordy CW, Sullivan ME. 2019 Distributed biological observatory region 1: physics, chemistry and plankton in the northern Bering sea. Deep-Sea Res. II Top. Stud. Oceanogr. **162**, 8–21. (10.1016/j.dsr2.2018.11.006)

[68] Hunt Jr GL, Stabeno P, Walters G, Sinclair E, Brodeur RD, Napp JM, Bond NA. 2002 Climate change and control of the southeastern Bering sea pelagic ecosystem. Deep-Sea Res. II Top. Stud. Oceanogr. **49**, 5821–5853. (10.1016/S0967-0645(02)00321-1)

[69] Sigler MF, Stabeno PJ, Eisner LB, Napp JM, Mueter FJ. 2014 Spring and fall phytoplankton blooms in a productive subarctic ecosystem, the Eastern Bering sea, during 1995–2011. Deep-Sea Res. II Top. Stud. Oceanogr. **109**, 71–83. (10.1016/j.dsr2.2013.12.007)

[70] Eisner LB, Napp JM, Mier KL, Pinchuk AI, Andrews AG III. 2014 Climate-mediated changes in zooplankton community structure for the Eastern Bering sea. Deep-Sea Res. II Top. Stud. Oceanogr. **109**, 157–171. (10.1016/j.dsr2.2014.03.004)

[71] Lovvorn JR, Cooper LW, Brooks ML, De Ruyck CC, Bump JK, Grebmeier JM. 2005 Organic matter pathways to zooplankton and benthos under pack ice in late winter and open water in late summer in the north-central Bering sea. Mar. Ecol. Prog. Ser. **291**, 135–150. (10.3354/meps291135)

[72] Lindström Å, Gill RE, Jamieson SE, McCaffery B, Wennerberg L, Wikelski M, Klaassen M. 2011 A puzzling migratory detour: are fueling conditions in Alaska driving the movement of juvenile sharp-tailed sandpipers? Condor **113**, 129–139. (10.1525/cond.2011.090171)

[73] Durell S. 2000 Individual feeding specialisation in shorebirds: population consequences and conservation implications. Biol. Rev. **75**, 503–518. (10.1111/j.1469-185X.2000.tb00053.x) 11117199

[74] Lovvorn JR, North CA, Kolts JM, Grebmeier JM, Cooper LW, Cui X. 2016 Projecting the effects of climate-driven changes in organic matter supply on benthic food webs in the northern Bering sea. Mar. Ecol. Prog. Ser. **548**, 11–30. (10.3354/meps11651)

[75] Van Colen C, Debusschere E, Braeckman U, Van Gansbeke D, Vincx M. 2012 The early life history of the clam Macoma balthica in a high CO_2_ world. PLoS One **7**, e44655. (10.1371/journal.pone.0044655) 22970279 PMC3438177

[76] Menu S, Gauthier G, Reed A. 2005 Survival of young greater snow geese (Chen caerulescens atlantica). Auk **122**, 479–496. (10.1093/auk/122.2.479)

[77] Siddon EC, Zador SG, Hunt GL. 2020 Ecological responses to climate perturbations and minimal sea ice in the northern Bering sea. Deep Sea Res. II Top. Stud. Oceanogr. **181–182**, 104914. (10.1016/j.dsr2.2020.104914)

[78] Grebmeier JM *et al*. 2006 A major ecosystem shift in the northern Bering sea. Science **311**, 1461–1464. (10.1126/science.1121365) 16527980

[79] Rakhimberdiev E, van den Hout PJ, Brugge M, Spaans B, Piersma T. 2015 Seasonal mortality and sequential density dependence in a migratory bird. J. Avian Biol. **46**, 332–341. (10.1111/jav.00701)

[80] Newton I. 2006 Can conditions experienced during migration limit the population levels of birds? J. Ornithol. **147**, 146–166. (10.1007/s10336-006-0058-4)

[81] Senner NR, Morbey YE, Sandercock BK. 2020 Editorial: flexibility in the migration strategies of animals. Front. Ecol. Evol. **8**, 111. (10.3389/fevo.2020.00111)

[82] Zimova M, Willard DE, Winger BM, Weeks BC. 2021 Widespread shifts in bird migration phenology are decoupled from parallel shifts in morphology. J. Anim. Ecol. **90**, 2348–2361. (10.1111/1365-2656.13543) 34151433

[83] Shamoun-Baranes J, Leyrer J, van Loon E, Bocher P, Robin F, Meunier F, Piersma T. 2010 Stochastic atmospheric assistance and the use of emergency staging sites by migrants. Proc. Biol. Sci. **277**, 1505–1511. (10.1098/rspb.2009.2112) 20071381 PMC2871836

[84] Chernetsov NS. 2016 Orientation and navigation of migrating birds. Biol. Bull. Russ. Acad. Sci. **43**, 788–803. (10.1134/S1062359016080069)

[85] Sergio F, Tanferna A, De Stephanis R, Jiménez LL, Blas J, Tavecchia G, Preatoni D, Hiraldo F. 2014 Individual improvements and selective mortality shape lifelong migratory performance. Nature **515**, 410–413. (10.1038/nature13696) 25252973

[86] Verhoeven MA, Loonstra AHJ, McBride AD, Kaspersma W, Hooijmeijer J, Both C, Senner NR, Piersma T. 2022 Age‐dependent timing and routes demonstrate developmental plasticity in a long‐distance migratory bird. J. Anim. Ecol. **91**, 566–579. (10.1111/1365-2656.13641) 34822170 PMC9299929

[87] Owen M, Black JM. 1989 Factors affecting the survival of barnacle geese on migration from the breeding grounds. J. Anim. Ecol. **58**, 603. (10.2307/4851)

[88] Anderson AM, Duijns S, Smith PA, Friis C, Nol E. 2019 Migration distance and body condition influence shorebird migration strategies and stopover decisions during southbound migration. Front. Ecol. Evol. **7**, 251. (10.3389/fevo.2019.00251)

[89] Gill RE *et al*. 2009 Extreme endurance flights by landbirds crossing the Pacific ocean: ecological corridor rather than barrier? Proc. Biol. Sci. **276**, 447–457. (10.1098/rspb.2008.1142) 18974033 PMC2664343

[90] Ruthrauff DR, Tibbitts TL, Gill Jr RE, Handel CM. 2023 USGS Alaska science center adult shorebird morphological measurement data. US Geol. Surv. Data Release (10.5066/P9KNRWXB)

[91] Lagassé BJ, Breed GA. 2024 Supplementary material, data, and code for: warming sea surface temperatures are linked to lower shorebird migratory fuel loads.Figshare. (10.6084/m9.figshare.24295009) PMC11252674 39021777

